# Disaccharidase Inhibitory Activity of Thai Plant Extracts

**DOI:** 10.3390/nu18030456

**Published:** 2026-01-30

**Authors:** Masashi Kawami, Ryoko Yumoto, Varaporn Buraphacheep Junyaprasert, Noppamas Soonthornchareonnon, Denpong Patanasethanont, Bungorn Sripanidkulchai, Mikihisa Takano

**Affiliations:** 1 Department of Pharmaceutics, School of Pharmaceutical Sciences, Mukogawa Women’s University, Kyubanmachi, Koshien, Nishinomiya 668-8179, Japan; 2 Department of Pharmaceutics and Therapeutics, Graduate School of Biomedical Sciences, Hiroshima University, 1-2-3 Kasumi, Minami-ku, Hiroshima 734-8553, Japan; ryumoto@hiroshima-u.ac.jp; 3 Faculty of Pharmacy, Mahidol University, 447 Sri-Ayutthaya, Rajathavee, Bangkok 10400, Thailand; varaporn.jun@mahidol.ac.th (V.B.J.); noppamas.sup@mahidol.ac.th (N.S.); 4 Faculty of Pharmaceutical Sciences, Khon Kaen University, Khon Kaen 40002, Thailand; denpat@kku.ac.th (D.P.); bungorn@kku.ac.th (B.S.); 5 Department of Pharmaceutics, Faculty of Pharmacy, Yasuda Women’s University, 6-13-1 Yasuhigashi, Asaminami-ku, Hiroshima 731-0153, Japan

**Keywords:** enzyme kinetics, ethnopharmacology, intestinal disaccharidases, postprandial hyperglycemia, traditional Thai medicinal plants

## Abstract

**Background/Objectives**: Intestinal α-glucosidases, including maltase, sucrase, and trehalase, are key enzymes responsible for the final steps of carbohydrate digestion. Although Thai medicinal plants possess diverse bioactivities, most previous studies on plant-derived α-glucosidase inhibitors have focused on single-enzyme assays, primarily maltase, and lack systematic comparison of the three major intestinal disaccharidases—maltase, sucrase, and trehalase. This study aimed to determine the kinetic properties of rat intestinal α-glucosidases and evaluate the inhibitory potential of selected Thai plant extracts. **Methods**: Rat small-intestinal S9 fractions, post-mitochondrial supernatant obtained by centrifugation at 9000× *g*, containing soluble enzymes and microsomal components responsible for disaccharidase activity, were prepared and disaccharidase activities were quantified using the glucose oxidase–peroxidase method. Kinetic parameters were obtained from Eadie–Hofstee plots using maltose, sucrose, and trehalose as substrates. Fourteen Thai plant extracts (*Oryza sativa*, *Cratoxylum formosum*, *Garcinia cawa*, *Aganosma marginata*, *Polyalthia evecta*, *Ellipeiopsis cherrevensis*, *Ancistrocladus tectorius*, *Micromelum minutum*, and *Microcos tomentosa*) and isolated compounds (Bergapten, Eurycomalactone, Lupinifolin, Osthole) were assessed at 100 and 250 µg/mL for inhibition of maltase, sucrase, and trehalase. **Results**: Maltase exhibited the highest substrate affinity based on the lowest Km value. Among the tested samples, the 80% ethanol extract of *Microcos tomentosa* (MT80) inhibited maltase, sucrase, and trehalase activities by approximately 10–60% at 250 µg/mL, and was the only extract showing consistent inhibition across all three enzymes. Other extracts showed selective inhibition toward one or two enzymes. **Conclusions**: These findings indicate that MT80 possesses a broad-spectrum inhibitory profile against major intestinal α-glucosidases, suggesting a potential advantage for comprehensive regulation of postprandial glucose excursions and supporting its candidacy as a source of novel α-glucosidase inhibitors.

## 1. Introduction

Postprandial hyperglycemia is a key therapeutic target in the management of type 2 diabetes mellitus, because repeated glucose excursions after meals contribute to the development of microvascular and macrovascular complications [1]. Intestinal α-glucosidases located in the brush border membrane of enterocytes hydrolyze oligosaccharides and disaccharides, such as maltose, sucrose and trehalose, into glucose [2], thereby determining the rate of glucose appearance in the circulation. Pharmacological inhibition of these enzymes is therefore an established strategy to blunt postprandial glucose spikes as demonstrated by systematic reviews and meta-analyses evaluating α-glucosidase-inhibiting drugs [3].

Currently, only three α-glucosidase inhibitors—acarbose, voglibose and miglitol—are approved for clinical use as oral antidiabetic agents. These drugs reduce postprandial glucose increments and improve glycemic control; however, their broader application is often limited by gastrointestinal adverse effects, including flatulence, abdominal distension and diarrhea, which arise from undigested carbohydrates reaching the colon and being fermented by the gut microbiota [4,5]. Thus, there remains an unmet need for novel α-glucosidase inhibitors with comparable or superior efficacy but improved tolerability profiles.

Natural products of plant origin have long been recognized as an important source of antidiabetic agents, and numerous plant-derived molecules with α-glucosidase inhibitory activity have been identified over the past decades [6,7]. Recent comprehensive reviews of plant-based α-glucosidase inhibitors have highlighted structurally diverse classes such as flavonoids, stilbenes, tannins and triterpenoids, some of which exhibit inhibitory potencies comparable to or greater than acarbose in vitro [8], suggesting that they may serve as promising leads for the development of new postprandial glucose-lowering drugs. In line with these findings, traditional herbal medicines used for the treatment of diabetes in various regions have attracted increasing attention as reservoirs of bioactive α-glucosidase inhibitors.

Thailand has a rich tradition of herbal remedies for metabolic diseases, and several Thai medicinal formulas and constituent plants have recently been shown to possess α-glucosidase inhibitory activity in vitro and in vivo [9]. For example, extracts prepared from Thai folk antidiabetic remedies and Thai culinary or medicinal plants have been reported to inhibit α-glucosidase with IC_50_ values comparable to, or lower than, those of acarbose, and in some cases to improve glucose tolerance in experimental models [10,11]. In more recent studies, ten Thai culinary vegetables were shown to inhibit not only α-glucosidase but also α-amylase in vitro [12], and the traditional Thai remedy Krom Luang Chumphon Khet Udomsak exhibited significant α-glucosidase inhibitory activity supported by molecular docking analysis [13]. These observations support the concept that Thai ethnomedicinal plants may harbor compounds suitable for the development of novel α-glucosidase inhibitors.

Despite the growing evidence on plant-derived α-glucosidase inhibitors, most previous studies have focused on single-enzyme screening assays, predominantly targeting maltase or total α-glucosidase activity, without considering enzyme-specific differences among maltase, sucrase, and trehalase [4,14]. However, these intestinal disaccharidases differ substantially in substrate specificity, kinetic properties, and physiological roles in carbohydrate digestion [15], suggesting that selective or broad-spectrum inhibition may lead to distinct metabolic outcomes. In particular, trehalase has received comparatively little attention, despite the increasing dietary exposure to trehalose and its emerging relevance to metabolic health [16,17]. Consequently, a comprehensive evaluation of enzyme-specific inhibition across major intestinal disaccharidases is required to better understand the pharmacological potential of plant-derived α-glucosidase inhibitors in a global nutritional context.

So far, we have focused on the *p*-glycoprotein (*p*-gp)–inhibitory effects of Thai plant extracts and demonstrated their ability to reverse multidrug resistance both in vitro and in vivo [18,19]. We further showed that several of these extracts could resensitize *p*-gp–overexpressing cancer cells to anticancer agents through potent inhibition of *p*-gp–mediated drug efflux [20]. These findings highlight the capacity of Thai medicinal plants to modulate key intestinal functions that are relevant not only to drug disposition but also to nutrient handling. Despite this, the influence of these Thai medicinal plants on carbohydrate-digesting enzymes, particularly intestinal α-glucosidases, has not yet been elucidated. In the present study, we therefore characterized the kinetic properties of rat intestinal α-glucosidases using maltose, sucrose, and trehalose as substrates, and subsequently conducted a systematic evaluation of the inhibitory effects of a panel of Thai plant extracts and related compounds on maltase, sucrase, and trehalase activities in rat small intestinal S9 fractions. To our knowledge, this is the first study to provide a comparative kinetic and inhibitory profiling of all three major intestinal α-glucosidases simultaneously, including trehalase, whose physiological relevance is increasing in parallel with rising dietary trehalose intake.

## 2. Materials and Methods

### 2.1. Plant Materials

Ethanol extracts and purified compounds used in this study are listed in Table 1. The 50% ethanol extracts KP006 (*Cratoxylum formosum*), KP007 (*Garcinia cowa*), KP008 (*Aganosma marginata*), KP011 (*Polyalthia evecta*), KP018 (*Ellipeiopsis cherrevensis*), and RC-01 (*Oryza sativa*) were kindly provided by Khon Kaen University, Thailand. The 80% ethanol extracts AT80 (*Ancistrocladus tectorius*), MM80 (*Micromelum minutum*), and MT80 (*Microcos tomentosa*) were gifts from Mahidol University, Thailand. Preparation procedures of these ethanol extracts were described previously [18]. Briefly, dried and powdered plant materials were extracted with 50% or 80% ethanol at room temperature, followed by filtration and solvent removal under reduced pressure. The resulting extracts were evaporated to dryness and stored at −20 °C until use.

Four purified compounds—bergapten (BER), osthole (OST), eurycomalactone (ECL), and lupinifolin (LUP)—were also obtained from Mahidol University. Their isolation and purification methods, including solvent extraction and chromatographic procedures, were performed as previously reported [18]. All extracts were evaporated to dryness under reduced pressure and stored at −20 °C until use.

DMSO was used as a solvent at a final concentration of 0.5% (*v*/*v*) in all experimental conditions, including controls. This concentration of DMSO did not affect cell viability, as confirmed by XTT assays (Supplementary Figure S1).

### 2.2. Animals

Male Wistar rats (5–7 weeks) were purchased from Japan SLC, Inc. (Shizuoka, Japan). Upon arrival, the animals were housed in a temperature-controlled room maintained at 25 °C. The rats were fasted with free access to water and used for experiments within 24 h after arrival, without a prolonged acclimatization period.

### 2.3. Isolation of Rat S9 Fraction

Preparation of rat intestinal S9 fraction, post-mitochondrial supernatant obtained by centrifugation at 9000× *g*, containing soluble enzymes and microsomal components responsible for disaccharidase activity, was performed in accordance with the Guide for Animal Experimentation from Hiroshima University and the guidelines of the Committee of Research Facilities for Laboratory Animal Sciences, Hiroshima University (approval number: A09–010, 4 April 2009). Rat intestinal S9 fractions were prepared from male rats under pentobarbital anesthesia. To minimize variability in disaccharidase activity along the intestinal axis, approximately 10 cm distal to the stomach and 10 cm proximal to the cecum were excluded. The remaining middle portion of the small intestine, comprising both the jejunum and ileum, was used for S9 fraction preparation. The isolated segment was inverted, and the mucosal layer was gently scraped using a coverslip. The collected mucosa was weighed and suspended in nine volumes of ice-cold PBS(–), a phosphate-buffered saline composed of 137 mM NaCl, 2.7 mM KCl, 8.1 mM Na_2_HPO_4_, and 1.5 mM KH_2_PO_4_ (pH 7.4). The suspension was homogenized and centrifuged at 9000× *g* for 20 min at 4 °C, and the supernatant was collected as the S9 fraction. Protein concentrations were determined by the Lowry method [18] using bovine serum albumin as the standard, and the S9 fraction was diluted appropriately (10–20-fold) for enzymatic assays. Approximately 20 mL of S9 fraction was obtained from each animal. The protein concentrations of the S9 fractions were 7.79 mg/mL (rat 1), 7.52 mg/mL (rat 2), and 7.37 mg/mL (rat 3), respectively. Each S9 fraction prepared from a single animal was used independently for enzymatic assays. Thus, three independent experiments correspond to S9 fractions obtained from three individual rats.

### 2.4. Measurement of Disaccharidase Activity Using the Glucose Oxidase Method

Disaccharidase activities were determined using maltose, sucrose, and trehalose as substrates. Rat intestinal S9 fractions were diluted to final protein concentrations of 0.5 mg/mL (maltase), 7 mg/mL (sucrase), and 3 mg/mL (trehalase). These protein concentrations were determined based on preliminary experiments to ensure sufficient glucose production and linear reaction kinetics for each disaccharidase. Because the intrinsic activities and expression levels of maltase, sucrase, and trehalase differ in rat intestinal S9 fractions, different protein concentrations were required to obtain reliable and comparable measurements of enzymatic activity. Plant extracts were dissolved in DMSO and added to the reaction mixture at a final concentration of 0.5%. The diluted S9 fractions were preincubated at 37 °C for 10 min, followed by incubation with each substrate for 30 min at 37 °C. Reactions were terminated by heating at 90–100 °C for 10 min.

After cooling, samples were centrifuged at 10,000 rpm for 10 min, and supernatants were subjected to glucose quantification. The colorimetric reagent consisted of 1.5 U/mL peroxidase (75 U in 50 mL, FUJIFILM Wako Pure Chemical Corporation (Osaka, Japan)), 2 U/mL glucose oxidase (100 U in 50 mL, FUJIFILM Wako Pure Chemical Corporation), and 0.12 M sodium phosphate buffer (pH 7.4) to a final volume of 50 mL. To this mixture, 0.5 mL of 9 mM o-dianisidine solution was added, yielding 50.5 mL of the final chromogenic reagent. After mixing the reagent with each sample, the reaction proceeded for 40 min at room temperature and was stopped by adding 70% H_2_SO_4_. Absorbance was measured at 530 nm, and glucose standards were used for calibration.

### 2.5. Calculation of Enzymatic Kinetic Parameters

Kinetic parameters of intestinal disaccharidases were determined using glucose production rates obtained at multiple substrate concentrations. Maltase activity was measured at maltose concentrations of 0 (control), 1.25, 2.5, 5, 10, 25, and 50 mM, whereas sucrase and trehalase activities were assessed at sucrose or trehalose concentrations of 0 (control), 2.5, 5, 10, 20, 50, and 100 mM. The glucose production rate (V, nmol/mg protein/min) at each substrate concentration (S) was fitted to the Michaelis–Menten equation. Data were further transformed to Eadie–Hofstee plots (V vs. V/S), which are commonly used in enzyme kinetics to evaluate Michaelis–Menten behavior and confirm single-enzyme–mediated substrate hydrolysis, and linear regression was applied. The negative slope of the regression line was taken as the Km value, and the y-intercept was used to obtain Vmax. Residual enzyme activity was expressed as a percentage of control activity and calculated using the following equation:

Residual activity (%) = (OD_{530 nm, sample}/OD_{530 nm, control}) × 100.

Enzyme activity in the absence of plant extracts or compounds was defined as 100%.

### 2.6. Statistical Analysis

All data are expressed as the mean ± standard error of the mean (SEM) from three independent experiments (*n* = 3 biological replicates corresponding to S9 fractions prepared from different rats). Statistical significance was evaluated using one-way analysis of variance (ANOVA) followed by Tukey’s post hoc test for multiple comparisons. A *p* value of less than 0.05 was considered statistically significant.

## 3. Results

### 3.1. Basal Disaccharidase Activities in Rat Intestinal S9 Fractions

**Figure 1:**
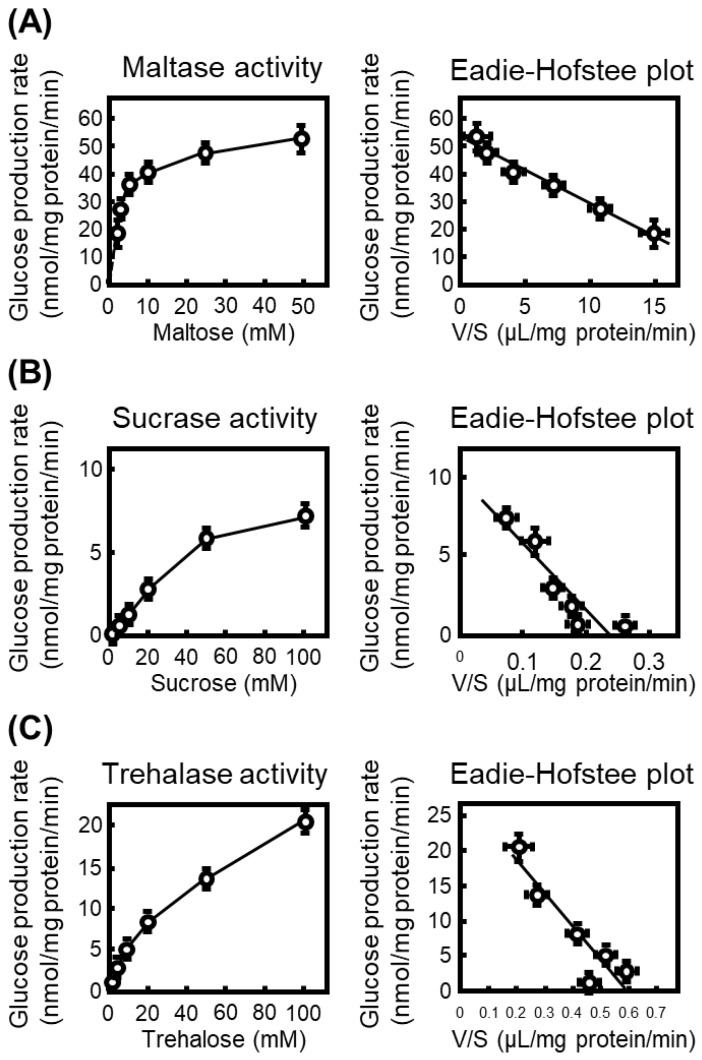
Kinetic characterization of intestinal disaccharidases in rat small-intestinal S9 fractions. (**A**) Maltase activity measured at increasing concentrations of maltose (1.25–50 mM). The glucose production rate (nmol/mg protein/min) was plotted against substrate concentration, and kinetic parameters were calculated from the corresponding Eadie–Hofstee plot with the ratio of reaction velocity (V) to substrate concentration (S). (**B**) Sucrase activity determined using sucrose (2.5–100 mM) as the substrate, with kinetic parameters obtained from Eadie–Hofstee transformation. (**C**) Trehalase activity measured at trehalose concentrations of 2.5–100 mM. All data represent mean ± SEM from three independent experiments (*n* = 3).

To characterize the intrinsic disaccharidase activities prior to inhibitor testing, we first quantified glucose production from maltose, sucrose, and trehalose using rat small-intestinal S9 fractions. Glucose formation increased in a substrate concentration-dependent manner for all three disaccharides, and kinetic parameters were obtained from Eadie–Hofstee plots converted from the Michaelis–Menten equation (Figure 1).

The calculated Km values were 2.3 mM (Vmax: 52.3 nmol/mg protein/min) for maltose, 41.9 mM (Vmax: 9.80 nmol/mg protein/min) for sucrose, and 47 mM (Vmax: 28.0 nmol/mg protein/min) for trehalose, indicating that maltase exhibits the highest substrate affinity among the intestinal α-glucosidases examined. Accordingly, maltase activity was the most prominent in rat intestinal S9 fractions.

### 3.2. Effect of Thai Plant Extracts and Purified Compounds on Disaccharidase Activities

To exclude the possibility that the observed reduction in residual disaccharidase activity was due to non-specific cytotoxic effects, the viability of HepG2 cells exposed to Thai plant extracts was evaluated. As shown in Supplementary Figure S1, treatment with each plant extract or isolated compound at 100 µg/mL for 24 h did not significantly affect cell viability compared with the vehicle control (0.5% DMSO). However, KP008 showed a reduction in cell viability to approximately 70% of control levels, although this decrease did not reach statistical significance. Therefore, the possibility that the observed inhibitory effect of KP008 reflects, at least in part, non-specific effects associated with cytotoxic constituents cannot be excluded. Overall, these results indicate that the concentrations used in the enzymatic assays were not associated with overt cytotoxicity and support the interpretation that the observed enzyme inhibition reflects specific effects on intestinal α-glucosidase activity rather than non-specific toxic effects.

Based on the Km values determined in Figure 1, inhibitory assays were performed at substrate concentrations of 2 mM for maltose, 40 mM for sucrose, and 50 mM for trehalose. Each plant extract or compound was examined at two concentrations (100 and 250 µg/mL), and the inhibitory ratios were calculated from the glucose formation rates.

**Figure 2:**
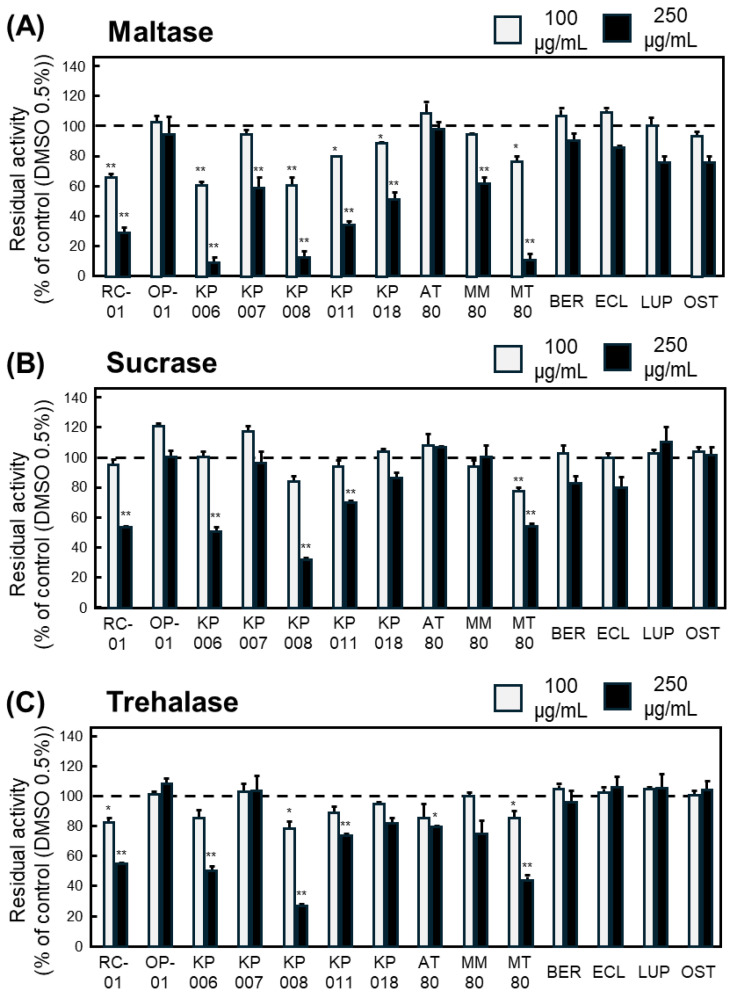
Inhibitory effects of Thai plant extracts and isolated compounds on intestinal α-glucosidase activities. Residual activity of maltase (**A**), sucrase (**B**), and trehalase (**C**) reduced by plant extracts and purified compounds at 100 (gray bar) and 250 (black bar) µg/mL. Enzyme activity is expressed as residual activity (%) relative to control (DMSO 0.5%) samples incubated without plant extracts or compounds, which were defined as 100% (a dashed line in each Figure). Data represents mean ± SEM from three independent experiments (*n* = 3). Statistical significance vs. control: * *p* < 0.05, ** *p* < 0.01.

For maltase, six samples—RC-01, KP006, KP008, KP011, KP018, and MT80—significantly inhibited enzymatic activity at both concentrations (Figure 2A). For sucrase, only MT80 exhibited significant inhibition at both concentrations (Figure 2B). For trehalase, RC-01, KP008, and MT80 significantly reduced glucose production in a concentration-independent manner (Figure 2C).

Collectively, MT80 was the only extract that consistently inhibited all three α-glucosidases, indicating that the 80% ethanol extract of *Microcos tomentosa* contains constituents with potent and broad α-glucosidase-inhibitory activity.

## 4. Discussion

In this study, we demonstrated that the 80% ethanol extract of MT80 exhibits a broad inhibitory effect on three major intestinal α-glucosidases—maltase, sucrase, and trehalase—using rat small-intestinal S9 fractions. To our knowledge, this is the first report showing that *M. tomentosa* possesses inhibitory activity against all three disaccharidases, indicating that this plant may serve as a promising source of multifunctional α-glucosidase inhibitors. In contrast, other Thai plant extracts tested in this study displayed selective inhibitory profiles that were limited to one or two enzymes, further highlighting the distinctive properties of MT80.

The intestinal α-glucosidase family comprises enzymes with different substrate specificities and structural features, and previous studies have suggested that inhibitors often differentiate between maltase, sucrase, and trehalase. The ability of MT80 to inhibit all three enzymes suggests that it contains bioactive constituent(s) capable of targeting conserved regions or shared catalytic features of these disaccharidases. *M. tomentosa* is known to contain triterpenoids, sterols, and other secondary metabolites [21,22], some of which share structural characteristics with previously reported plant-derived α-glucosidase inhibitors [4,23]. Polyphenolic compounds are known to interact with catalytic residues or substrate-binding subsites of α-glucosidases, often through hydrogen bonding and π–π interactions, whereas terpenoid constituents have been reported to bind to peripheral or conserved structural domains and exert non-competitive or mixed-type inhibition [4,23]. Alkaloid-like structures, when present, may further interact with conserved catalytic motifs shared among glycoside hydrolases, potentially contributing to enzyme inhibition across different α-glucosidase isoforms [24]. Although the present study did not identify individual active constituents or experimentally evaluate synergistic effects, it is plausible that the combination of these structurally diverse compounds in MT80 contributes to simultaneous inhibition of multiple intestinal α-glucosidases by targeting conserved catalytic or structural features. Such cooperative or additive effects have been frequently suggested in studies reporting stronger inhibitory activity of crude plant extracts compared with isolated single compounds. Further studies using purified constituents and combination analyses will be required to clarify the precise molecular basis of the broad-spectrum inhibitory activity of MT80.

Numerous plant-derived α-glucosidase inhibitors have been reported [23]. However, a common methodological feature of many of these studies is the reliance on single-enzyme screening assays, most frequently targeting maltase or total α-glucosidase activity, without discriminating between individual intestinal disaccharidases [4]. Comprehensive enzyme-specific evaluations that simultaneously assess maltase, sucrase, and trehalase activities remain relatively limited. In East Asian studies, particularly those focusing on traditional Chinese and Korean medicinal plants, α-glucosidase inhibitory activity has frequently been associated with flavonoid- and polyphenol-rich secondary metabolites. For example, phytochemical investigations of *Viburnum* species have identified diverse phenolic compounds exhibiting α-glucosidase inhibitory activity, which has predominantly been evaluated using in vitro screening assays such as yeast α-glucosidase models [25,26]. Additionally, several plant extracts from Southeast Asia including Indonesia, have been discovered to have the potential to inhibit α-glucosidase activity [27]. The extract of *Psiadia punctulata*, mostly found in some East African countries, including Eritrea, Saudi Arabia, and North East India, shows clear inhibitory effect on the conversion of maltose into glucose [28]. Although these studies have provided valuable insights into the inhibitory potential of the plant extract against α-glucosidases, enzyme-specific differences among intestinal disaccharidases have generally not been addressed. In this context, the present study differs from previous regional investigations by providing an enzyme-resolved analysis of intestinal disaccharidase inhibition. The observation that the 80% ethanol extract of *M. tomentosa* inhibited maltase, sucrase, and trehalase activities simultaneously contrasts with the predominantly selective inhibition reported for many plant extracts from other regions. This broad inhibitory profile suggests that MT80 may interact with conserved catalytic or structural features shared among intestinal α-glucosidases, highlighting its potential relevance beyond regional dietary or ethnomedical applications.

From a pharmacological perspective, broad-spectrum inhibition of intestinal disaccharidases may be advantageous for regulating postprandial glucose excursions, a key therapeutic target in the management of type 2 diabetes [23]. Clinically used α-glucosidase inhibitors such as acarbose primarily inhibit maltase and sucrase, with limited effects on trehalase [29]. In this context, the inhibitory profile of MT80—particularly its unique ability to inhibit trehalase—may offer an additional therapeutic benefit, especially in diets that include trehalose-rich foods such as mushrooms [16]. In contrast, trehalose shows therapeutic effect on obesity and metabolic disease [17], indicating that additional research addressment concerning clinical benefit of trehalase inhibition should be required.

We previously examined the effects of Thai plant extracts/compounds on *p*-gp activity in cancer cell lines [18,20], and found that MT80 had no effect on the accumulation of paclitaxel in *p*-gp-overexpressing cancer cells [18]. *p*-gp substrates such as paclitaxel refer to compounds that are actively transported by *p*-gp, which leads to limitation of intestinal absorption and drug–drug interaction. This finding indicates that MT80 would not interact with *p*-gp function in the intestine. Avoiding interaction with intestinal *p*-gp not only reduces the risk of drug–drug interactions with other *p*-gp substrate medications but also helps maintain the physiological efflux capacity for xenobiotic elimination.

In addition to MT80, certain Thai plant extracts exhibited enzyme-selective inhibitory profiles. Notably, KP006 (*C. formosum*) showed relatively strong inhibitory activity against maltase, whereas little or no inhibition was observed toward sucrase or trehalase. Such maltase-selective inhibition is mechanistically plausible, as previous studies have demonstrated that specific classes of polyphenols and xanthone derivatives preferentially inhibit maltase–glucoamylase over other intestinal disaccharidases, likely due to differences in substrate-binding pockets and catalytic domain architecture [4,23]. *C. formosum* is known to contain xanthones and related phenolic compounds [30,31], suggesting that these constituents may contribute to the enzyme-specific effects observed for KP006. In contrast, MT80 uniquely inhibited maltase, sucrase, and trehalase activities simultaneously, indicating a broader interaction with conserved catalytic or structural features shared among intestinal α-glucosidases, and thus may be more advantageous for comprehensive modulation of postprandial carbohydrate digestion.

KP008 exhibited apparent inhibitory activity against maltase; however, Supplementary Figure S1 shows a trend toward reduced HepG2 cell viability following KP008 treatment, although the difference did not reach statistical significance. Therefore, it cannot be excluded that the observed reduction in enzyme activity reflects, at least in part, non-specific effects associated with cytotoxic constituents rather than selective inhibition of intestinal disaccharidases. Consistent with this interpretation, Khay M et al. [32] have reported that methanol extract of *Aganosma marginata*, the source plant of KP008, showed pronounced cytotoxic or antiproliferative properties in HepG2 and HT29 colon adenocarcinoma cells. Accordingly, further studies using purified constituents and lower, non-cytotoxic concentration ranges will be required to clarify whether KP008 exerts genuine α-glucosidase–specific inhibition. Taken together, while KP006 represents a potentially useful source of maltase-selective inhibitors, the broad-spectrum inhibitory profile of MT80, together with its lower likelihood of non-specific toxicity, positions it as the most promising candidate among the extracts examined in this study.

Several limitations should be acknowledged. The present study did not identify the specific active compounds in MT80 responsible for enzyme inhibition, and in vivo efficacy remains to be evaluated. Nevertheless, the clear inhibitory profile observed across all three disaccharidases provides a strong rationale for future studies focusing on bioactive compound isolation and in vivo validation. Taken together, our findings indicate that *Microcos tomentosa* contains constituents with potent and broad inhibitory activity toward intestinal α-glucosidases, positioning MT80 as a promising candidate for the development of new α-glucosidase inhibitors for postprandial glucose control.

## 5. Conclusions

In summary, the present study demonstrates that MT80, 80% ethanol extract of *Microcos tomentosa*, exhibits potent and broad inhibitory activity toward intestinal disaccharidases, including maltase, sucrase, and trehalase, in rat small-intestinal S9 fractions. Among the Thai plant extracts examined, MT80 was the only sample that consistently inhibited all three α-glucosidases, suggesting that this plant contains bioactive constituent(s) with multifunctional inhibitory potential. These findings highlight *M. tomentosa* as a promising candidate for the development of novel α-glucosidase inhibitors that may contribute to the control of postprandial glucose excursions.

As this study represents an initial enzyme-based evaluation, several important issues remain to be addressed in future investigations. First, identification and isolation of the bioactive constituents responsible for the observed inhibitory effects are required to clarify the structure–activity relationships underlying the broad-spectrum inhibition. Second, in vivo studies will be necessary to determine whether the enzyme-specific inhibitory profile of MT80 translates into effective modulation of postprandial glucose excursions under physiological conditions. In addition, potential interactions with dietary carbohydrates and intestinal transporters should be explored to better understand the nutritional and pharmacological relevance of MT80 in a global dietary context. Collectively, these future studies will help to further elucidate the therapeutic potential of *Microcos tomentosa* and contribute to the rational development of plant-derived α-glucosidase inhibitors with improved efficacy and tolerability. In addition, future studies should examine whether bioactive constituents derived from Microcos tomentosa can act synergistically or additively with clinically used α-glucosidase inhibitors, such as acarbose, to enhance glucose-lowering efficacy while potentially reducing gastrointestinal adverse effects. Such combination approaches may provide a rational strategy for improving postprandial glycemic control.

## Figures and Tables

**Table 1:** List of Thai plant extracts and compounds.

Code	Plant	Family	Extract/Compound
RC-01	*Oryza sativa*	Gramineous	50% ethanol extract
KP006	*Cratoxylum formosum*	Clusiaceae	50% ethanol extract
KP007	*Garcinia cowa*	Clusiaceae	50% ethanol extract
KP008	*Aganosma marginata*	Apocynaceae	50% ethanol extract
KP011	*Polyalthia evecta*	Annonaceae	50% ethanol extract
KP018	*Ellipeiopsis cherrevensis*	Annonaceae	50% ethanol extract
AT80	*Ancistrocladus tectorius*	Ancistrocladaceae	80% ethanol extract
MM80	*Micromelum minutum*	Rutaceae	80% ethanol extract
MT80	*Microcos tomentosa*	Tiliaceae	80% ethanol extract
BER	*Cnidium monnieri*	Apiaceae	Bergapten
ECL	*Eurycoma longifolia*	Simaroubaceae	Eurycomalactone
LUP	*Derris reticulata*	Fabaceae	Lupinifolin
OST	*Cnidium monnieri*	Apiaceae	Osthole

## Data Availability

The data that support the findings reported herein are available from the corresponding author upon reasonable request due to privacy.

## Supplementary Materials

The following supporting information can be downloaded at: https://www.mdpi.com/article/10.3390/nu18030456/s1, Figure S1: The effect of Thai plant extracts and the purified compounds on the viability of HepG2 cells.

## Abbreviations

The following abbreviations are used in this manuscript:

*p*-gp	*p*-glycoprotein
PBS	Phosphate-buffered saline

## References

[1] Shibib L., Al-Qaisi M., Guess N., Miras A.D., Greenwald S.E., Pelling M., Ahmed A. (2024). Manipulation of Post-Prandial Hyperglycaemia in Type 2 Diabetes: An Update for Practitioners. Diabetes Metab. Syndr. Obes..

[2] Rose D.R., Chaudet M.M., Jones K. (2018). Structural Studies of the Intestinal α-Glucosidases, Maltase-glucoamylase and Sucrase-isomaltase. J. Pediatr. Gastroenterol. Nutr..

[3] Alssema M., Ruijgrok C., Blaak E.E., Egli L., Dussort P., Vinoy S., Dekker J.M., Robertson D.M. (2021). Effects of alpha-glucosidase-inhibiting drugs on acute postprandial glucose and insulin responses: A systematic review and meta-analysis. Nutr. Diabetes.

[4] Dirir A.M., Daou M., Yousef A.F., Yousef L.F. (2021). A Review of Alpha-Glucosidase Inhibitors from Plants as Potential Candidates for the Treatment of Type-2 Diabetes. Phytochem. Rev..

[5] Ghani U. (2015). Re-Exploring Promising α-Glucosidase Inhibitors for Potential Development into Oral Anti-Diabetic Drugs: Finding Needle in the Haystack. Eur. J. Med. Chem..

[6] Kumar S., Narwal S., Kumar V., Prakash O. (2011). α-Glucosidase Inhibitors from Plants: A Natural Approach to Treat Diabetes. Pharmacogn. Rev..

[7] Kumari S., Saini R., Bhatnagar A., Mishra A. (2024). Exploring Plant-Based Alpha-Glucosidase Inhibitors: Promising Contenders for Combatting Type-2 Diabetes. Arch. Physiol. Biochem..

[8] Eawsakul K., Panichayupakaranant P., Ongtanasup T., Warinhomhoun S., Noonong K., Bunluepuech K. (2021). Computational Study and In Vitro Alpha-Glucosidase Inhibitory Effects of Medicinal Plants from a Thai Folk Remedy. Heliyon.

[9] Chayarop K., Peungvicha P., Temsiririrkkul R., Wongkrajang Y., Chuakul W., Rojsanga P. (2017). Hypoglycaemic Activity of Mathurameha, a Thai Traditional Herbal Formula Aqueous Extract, and Its Effect on Biochemical Profiles of Streptozotocin-Nicotinamide-Induced Diabetic Rats. BMC Complement. Altern. Med..

[10] Sakulkeo O., Wattanapiromsakul C., Pitakbut T., Dej-adisai S. (2022). Alpha-Glucosidase Inhibition and Molecular Docking of Isolated Compounds from Traditional Thai Medicinal Plant, Neuropeltis Racemosa Wall. Molecules.

[11] Srisongkram T., Waithong S., Thitimetharoch T., Weerapreeyakul N. (2022). Machine Learning and In Vitro Chemical Screening of Potential α-Amylase and α-Glucosidase Inhibitors from Thai Indigenous Plants. Nutrients.

[12] Ratananikom K., Juntaree V., Wichaiyo W., Khunluek K., Premprayoon K., Kubola J. (2024). In Vitro Evaluation of α-glucosidase and α-Amylase Inhibition in Thai Culinary Vegetables. Scientifica.

[13] Limcharoen T., Chaniad P., Chonsut P., Punsawad C., Juckmeta T., Konyanee A., Rais I.R., Sangkaew S. (2024). Alpha-Glucosidase Inhibition, Antioxidant Activities, and Molecular Docking Study of Krom Luang Chumphon Khet Udomsak, a Thai Traditional Remedy. Adv. Pharmacol. Pharm. Sci..

[14] Lee B.H., Rose D.R., Lin A.H., Quezada-Calvillo R., Nichols B.L., Hamaker B.R. (2016). Contribution of the Individual Small Intestinal α-Glucosidases to Digestion of Unusual α-Linked Glycemic Disaccharides. J. Agric. Food Chem..

[15] Elferink H., Bruekers J.P.J., Veeneman G.H., Boltje T.J. (2020). A comprehensive overview of substrate specificity of glycoside hydrolases and transporters in the small intestine: “A gut feeling”. Cell. Mol. Life Sci..

[16] Chen A., Gibney P.A. (2023). Dietary Trehalose as a Bioactive Nutrient. Nutrients.

[17] Yeh Y.S., Evans T.D., Jeong S.J., Liu Z., Ajam A., Cosme C., Huang J., Peroumal D., Zhang X., Javaheri A. (2025). Assessing the Efficacy of the Natural Disaccharide Trehalose in Ameliorating Diet-Induced Obesity and Metabolic Dysfunction. Front. Nutr..

[18] Kawami M., Yumoto R., Nagai J., Junyaprasert V.B., Soonthornchareonnon N., Patanasethanont D., Sripanidkulchai B.O., Takano M. (2010). Effect of Thai Plant Extracts on P-Glycoprotein Function and Viability in Paclitaxel-Resistant HepG2 Cells. Drug Metab. Pharmacokinet..

[19] Kawami M., Yamada Y., Toshimori F., Issarachot O., Junyaprasert V.B., Yumoto R., Takano M. (2017). Effect of *Curcuma comosa* Extracts on the Functions of Peptide Transporter and P-Glycoprotein in Intestinal Epithelial Cells. Pharmazie.

[20] Kawami M., Yamada Y., Issarachot O., Junyaprasert V.B., Yumoto R., Takano M. (2018). P-Gp Modulating Effect of *Azadirachta indica* Extract in Multidrug-Resistant Cancer Cell Lines. Pharmazie.

[21] Kaennakam S., Sichaem J., Khumkratok S., Siripong P., Tip-pyang S. (2013). A new taraxerol derivative from the roots of *Microcos tomentosa*. Nat. Prod. Commun..

[22] Somwong P., Suttisri R., Amnuoypol S. (2017). Chemical Constituents of *Microcos tomentosa*. Chem. Nat. Compd..

[23] Kashtoh H., Baek K.H. (2022). Recent Updates on Phytoconstituent Alpha-Glucosidase Inhibitors: An Approach towards the Treatment of Type Two Diabetes. Plants.

[24] Singh G., Verma A.K., Kumar V. (2016). Catalytic properties, functional attributes and industrial applications of β-glucosidases. 3 Biotech.

[25] Chen J., Zhao Z.Y., Zhang X.H., Shao J.H., Zhao C.C. (2021). Recent Advance on Chemistry and Bioactivities of Secondary Metabolites from *Viburnum* Plants: An Update. Chem. Biodivers..

[26] Sancheti S., Sancheti S., Lee S.H., Lee J.E., Seo S.Y. (2011). Screening of Korean Medicinal Plant Extracts for α-Glucosidase Inhibitory Activities. Iran J. Pharm. Res..

[27] Benjamin M.A.Z., Mokhtar M.R.A., Iqbal M., Abdullah A., Azizah R., Sulistyorini L., Mah-fudh N., Zakaria Z.A. (2024). Medicinal plants of Southeast Asia with anti-α-glucosidase activity as potential source for type-2 diabetes mellitus treatment. J. Ethnopharmacol..

[28] Kidane Y., Bokrezion T., Mebrahtu J., Mehari M., Gebreab Y.B., Fessehaye N., Achila O.O. (2018). In Vitro Inhibition of α-Amylase and α-Glucosidase by Extracts from *Psiadia punctulata* and *Meriandra bengalensis*. Evid.-Based Complement Altern. Med..

[29] Samulitis B.K., Goda T., Lee S.M., Koldovský O. (1987). Inhibitory mechanism of acarbose and 1-deoxynojirimycin derivatives on carbohydrases in rat small intestine. Drugs Exp. Clin. Res..

[30] Boonnak N., Karalai C., Chantrapromma S., Ponglimanont C., Kanjana-Opas A., Chan-trapromma K., Kato S. (2010). Chromene and prenylated xanthones from the roots of *Cratoxylum formosum* ssp. *pruniflorum*. Chem. Pharm. Bull..

[31] Xiong J., Liu X.H., Bui V.B., Hong Z.L., Wang L.J., Zhao Y., Fan H., Yang G.X., Hu J.F. (2014). Phenolic constituents from the leaves of *Cratoxylum formosum* ssp. *pruniflorum*. Fitoterapia.

[32] Khay M., Toeng P., Mahiou-Leddet V., Mabrouki F., Sothea K., Ollivier E., Elias R., Bun S.S. (2012). HPLC analysis and cytotoxic activity of *Vernonia cinerea*. Nat. Prod. Commun..

