# Supplementary material for: Disaccharidase Inhibitory Activity of Thai Plant Extracts
Source: Nutrients. 2026 Jan 30;18(3):456. doi: 10.3390/nu18030456 (PMC12899519; doi:10.3390/nu18030456)
Supplement: Supplementary file 1 [file nutrients-18-00456-s001.zip › nutrients-4036623-supplementary.pptx]

## Slide 1
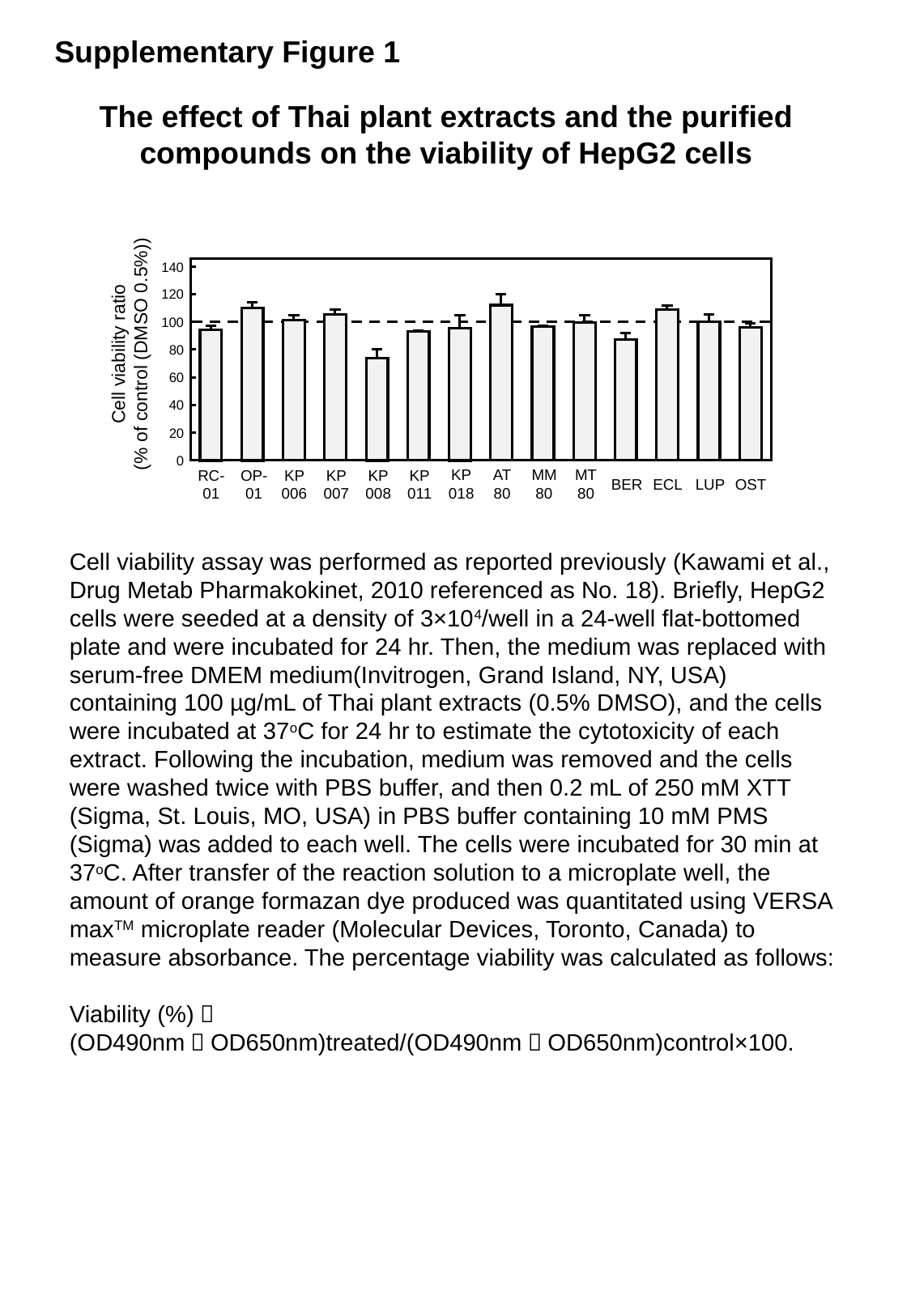

Supplementary Figure 1
The effect of Thai plant extracts and the purified compounds on the viability of HepG2 cells
140
120
100
Cell viability ratio
(% of control (DMSO 0.5%))
80
60
40
20
0
KP
018
AT
80
MM
80
MT
80
OP-01
KP
006
KP
007
KP
008
KP
011
RC-01
BER
ECL
OST
LUP
Cell viability assay was performed as reported previously (Kawami et al., Drug Metab Pharmakokinet, 2010 referenced as No. 18). Briefly, HepG2 cells were seeded at a density of 3×10^4^/well in a 24-well flat-bottomed plate and were incubated for 24 hr. Then, the medium was replaced with serum-free DMEM medium(Invitrogen, Grand Island, NY, USA) containing 100 µg/mL of Thai plant extracts (0.5% DMSO), and the cells were incubated at 37oC for 24 hr to estimate the cytotoxicity of each extract. Following the incubation, medium was removed and the cells were washed twice with PBS buffer, and then 0.2 mL of 250 mM XTT (Sigma, St. Louis, MO, USA) in PBS buffer containing 10 mM PMS (Sigma) was added to each well. The cells were incubated for 30 min at 37oC. After transfer of the reaction solution to a microplate well, the amount of orange formazan dye produced was quantitated using VERSA maxTM microplate reader (Molecular Devices, Toronto, Canada) to measure absorbance. The percentage viability was calculated as follows:
Viability (%)＝
(OD490nm－OD650nm)treated/(OD490nm－OD650nm)control×100.
